# Engaging Cytotoxic T and NK Cells for Immunotherapy in Chronic Lymphocytic Leukemia

**DOI:** 10.3390/ijms20174315

**Published:** 2019-09-03

**Authors:** Tom Hofland, Eric Eldering, Arnon P. Kater, Sanne H. Tonino

**Affiliations:** 1 Department of Experimental Immunology, Amsterdam Infection & Immunity Institute, Amsterdam UMC, University of Amsterdam, 1105 AZ Amsterdam, The Netherlands; 2 Department of Hematology, Cancer Center Amsterdam, Amsterdam UMC, University of Amsterdam, 1105 AZ Amsterdam, The Netherlands; 3 Lymphoma and Myeloma Center Amsterdam, LYMMCARE, 1105 AZ Amsterdam, The Netherlands

**Keywords:** chronic lymphocytic leukemia, T cells, natural killer cells, immunotherapy

## Abstract

Chronic lymphocytic leukemia (CLL) is characterized by an acquired immune dysfunction. CLL cells affect the phenotype and function of the entire spectrum of innate and adaptive immune cells, including monocytes, T cells, and natural killer (NK) cells, leading to a tumor-supportive environment and reduced immunosurveillance. Novel immunotherapies like immune checkpoint blockade, bi- and tri-specific antibodies, and chimeric antigen receptor (CAR) T cells use the patients’ immune system to induce therapeutic responses. Although these novel immunotherapies showed impressive results in several B cell lymphomas, responses in CLL were often disappointing. The strong immunomodulatory effect of CLL is believed to play a pivotal role in the low response rates to these immunotherapeutic strategies. In this review, we summarize how CLL influences the function of non-malignant lymphocytes, with a special focus on T and NK cells, two important cellular mediators for immunotherapy. Secondly, we provide a short overview of the activity of several immunotherapeutics in CLL, and discuss how novel strategies may overcome the disappointing response rates in CLL.

## 1. Introduction

Chronic lymphocytic leukemia (CLL) is a malignancy characterized by the accumulation of cluster of differentiation 19 (CD19)^+^ CD5^+^ B cells in blood, lymph nodes, and bone marrow [1]. CLL is the most common leukemia in the Western world, with approximately 20,000 new cases in the United States (US) each year [1]. The clinical course of the disease is heterogeneous among patients, but generally comprises a dormant phase in which patients do not require therapy, before the disease becomes more aggressive and treatment is needed. Until recently, first-line treatment for CLL mostly revolved around chemotherapeutic drugs which do not eradicate tumor cells completely, leading invariably to relapse of resistant disease [1]. Novel small-molecule inhibitors are changing frontline therapy in CLL. Agents like the Bruton’s tyrosine kinase (BTK) inhibitor ibrutinib and B-cell lymphoma 2 (Bcl-2) inhibitor venetoclax are highly effective in CLL and induce deep remissions, also in patients that relapse after chemotherapy [2,3]. However, both these agents require continuous treatment, and it is currently unclear whether treatment can be stopped at some point. This strategy harbors the risk of the development of escape mutations, which were already described for both ibrutinib and venetoclax [4,5]. Furthermore, lifelong treatment with these costly therapies leads to a high economic burden.

The only treatment for CLL that has curative potential is allogeneic hematopoietic stem-cell transplantation (allo-HSCT) [6]. Due to the advanced age and frailty of most CLL patients, transplantation is often not a realistic option. However, the observed graft-versus-leukemia effect indicates that the immune system harbors the potential for curing CLL patients.

Strategies to evoke autologous immune responses toward CLL cells can be divided into two main variants: antibody-mediated killing and direct cellular cytotoxicity. Mechanisms of the anti-tumor activity of treatment with monoclonal antibodies, such as the anti-CD20 antibodies rituximab, obinutuzumab, and ofatumumab, are a combination of direct induction of apoptotic signaling, complement activation, and induction of antibody-dependent cellular cytotoxicity via macrophages and natural killer (NK) cells [1,7]. On the other hand, direct cellular cytotoxicity is mediated mainly by cytotoxic lymphocytes like T and NK cells via the production of cytokines like interferon gamma (IFN-γ) and the direct induction of apoptosis via the release of cytotoxic granules or CD95/CD95-ligand (FAS/FAS-ligand) interaction. Newly developed immunotherapeutic strategies aim to induce these direct cytotoxic mechanisms for clinical responses.

**Figure 1:**
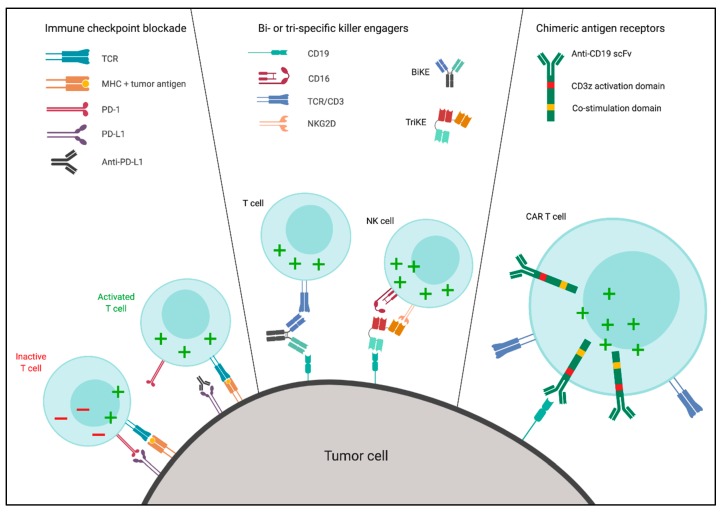
Overview of immunotherapeutic strategies. The left panel explains the mechanism of immune checkpoint blockade therapy, which prevents signaling by inhibitory receptors on cytotoxic lymphocytes, and unleashes tumor-specific immune responses. Programmed cell death protein 1/programmed death-ligand 1 (PD-1/PD-L1) blockade is given here as an example, but antibodies blocking cytotoxic T-lymphocyte-associated protein 4 (CTLA-4) were also approved, while other inhibitory receptors on T and natural killer (NK) cells may be targets for future strategies. The middle panel shows the mode of action of bi- and tri-specific killer engagers (BiKE and TriKE, respectively). Cytotoxic lymphocytes are recruited by one antibody arm of the construct, while the other arm binds to the tumor cell. In these examples, tumor cells are targeted via cluster of differentiation 19 (CD19), while T cells or NK cells are recruited via binding to CD3 or CD16 and NK cell receptor D (NKG2D), respectively. Due to the easy manufacturing of these constructs, many different strategies can be employed to target tumor cells and recruit effector cells. The right panel represents the use of chimeric antigen receptor (CAR) therapy, which redirects the specificity of autologous effector cells after CAR transduction via the extracellular single-chain variable fragment (scFv), and induces immune activation via intracellular activation and co-stimulation domains. Similar to the BiKE and TriKE constructs, CARs can be modified to target different tumor antigens. Furthermore, intracellular co-stimulation domains can vary, but most CARs contain CD28 or CD137 (4-1BB) co-stimulatory domains. Although the figure shows T cells as an example, NK cells can also be transduced with CAR constructs and used as effector cells. This figure was created using BioRender.

Immunotherapy using cytotoxic lymphocytes revolutionized cancer therapy, and several strategies also showed impressive efficacy in hematologic malignancies. Recruitment and activation of T and NK cells was achieved in multiple ways, which are summarized in Figure 1. Immune checkpoint blockade, which blocks signaling through inhibitory receptors on immune cells, induced clinical responses in several malignancies, most notably Hodgkin’s lymphoma [8,9]. Bi- and tri-specific antibody constructs, which recruit immune effector cells toward tumor cells by binding to both cell types simultaneously, target tumor cells for immune recognition, and showed activity in several non-Hodgkin lymphomas (NHL), like diffuse large B-cell lymphoma (DLBCL) and follicular lymphoma (FL) [10,11,12]. Finally, introducing chimeric antigen receptors (CAR) on the surface of cytotoxic cells redirects them toward tumor cells while simultaneously inducing immune activation, which was remarkably effective as a treatment in acute lymphoblastic leukemia (ALL) and, to a lesser extent, in NHL [13,14].

In contrast to the responses in other NHLs, clinical responses to these immunotherapies were relatively disappointing in CLL. Profound immune modulation by CLL is considered to be the explanation for the low response rates [15]. Several immune effector cells which are required for successful immunotherapy, like CD4^+^ and CD8^+^ T cells and natural killer (NK) cells, display phenotypic and functional defects in CLL.

In this review, we describe the known permutations in T and NK cells of CLL patients and how these changes affect the activity of immunotherapy with immune checkpoint inhibitors, bi- and tri-specific antibodies, and CAR constructs. Furthermore, we give an overview of immunotherapies that are currently under investigation for CLL and their effectivity, thereby providing an outlook on the future of CLL therapy.

## 2. Immunomodulation of Effector Cells in CLL

### 2.1. CD4^+^ and CD8^+^ T Cells

Studies on the immunomodulation by CLL mostly focused on changes in T-cell compartments (see Table 1). T-cell alterations in CLL were recognized for a long time, starting with the observation that both CD4^+^ and CD8^+^ T-cell numbers are increased in CLL patients [16,17]. The phenotype of T cells is markedly different in CLL patients compared to healthy individuals. Both CD4^+^ and CD8^+^ T cells in CLL show increased effector differentiation, with decreased numbers of naïve T cells and expansion of effector memory T-cell subsets [16,17,18,19,20]. Although part of these changes could be driven by antigen-specific expansion of T cells, CLL was also shown to influence T cells antigen-independently. The altered phenotype and function of both CD4^+^ and CD8^+^ T cells is influenced directly by CLL cells, as changes in gene expression can be induced in healthy T cells through co-culture with CLL cells [21,22]. 

The frequency of both T helper 1 and 2 (T_H_1 and T_H_2) CD4^+^ T cells is increased in CLL, and, although there is no clear T_H_1/T_H_2 skewing, T_H_2 expansion correlates with progressive disease, in line with their proposed tumor-supportive role in CLL [19,20]. Other tumor-supportive CD4^+^ T-cell subsets are also reported to be expanded in CLL. T follicular helper cells (T_fh_), which support CLL cell proliferation and survival via CD40L and the production of interleukin (IL)-21, are more frequent in blood and within lymph nodes of CLL patients, and their frequency increases with advanced disease [23,24,25]. Furthermore, regulatory T cells (T_reg_), which dampen anti-tumor immune responses, are expanded in blood and lymph nodes of CLL patients [26,27,28]. Interestingly, the frequency of T_reg_ cells is correlated with the tumor load, and co-culture of CLL cells with CD4^+^ T cells induces a forkhead box P3^+^ (FoxP3^+^) T_reg_ phenotype, indicating that CLL cells induce T_reg_ differentiation [26,29].

Despite the advanced effector differentiation state, CD8^+^ T cells show functional impairment in CLL, characterized by an inability to form immune synapses with target cells, decreased cytotoxicity, and reduced proliferation [21,22,30]. CD8^+^ T cells in CLL have elevated expression of several inhibitory receptors on the cell surface, like PD-1, CD160, CD244, and TIGIT (T cell immunoreceptor with Ig and ITIM domains), which were found to be involved in hampered immune synapse formation [23,30,31]. The increased expression of inhibitory receptors, coupled with the increase in CD8^+^ T-cell numbers, led to the belief that CD8^+^ T cells in CLL may be in a state of T-cell exhaustion, a process of gradual dysfunction due to chronic antigen exposure. However, since CD8^+^ T cells in CLL remain able to perform several functional responses, like the production of effector cytokines, “classical” T-cell exhaustion as described in solid tumors and chronic infection models probably does not apply to the CLL setting [30]. Functional impairment of CD8^+^ T cells can be induced in an antigen-independent manner via co-culture with CLL cells, showing that CLL cells affect CD8^+^ T cells via a different mechanism than chronic antigen stimulation [21,22]. Despite the fact that CLL alters functionality of CD8^+^ T cells outside the context of antigens, some CD8^+^ T-cell subsets are able to escape CLL-induced dysfunction, as cytomegalovirus (CMV)-specific CD8^+^ T cells were shown to be fully functional within the CLL micro-environment [18].

T-cell receptor (TCR) repertoires of CD4^+^ and CD8^+^ T cells in CLL show decreased diversity and skewed clonal expansion [32,33]. Expanded T-cell clones in CLL persist over time, and T-cell expansion correlates with tumor load, leading to the hypothesis that expanded T-cell clones contain tumor-specific T-cell populations [32,33]. Indeed, several reports describe the presence of antigen-specific T cells in CLL that respond to mutated peptides within tumor cells, and correlate with improved tumor control [34,35].

Taken together, although CD4^+^ and CD8^+^ T cells were shown to be able to recognize CLL tumor cells, functional modulation by the tumor clone leads to inhibition of T-cell-mediated immune responses and inadequate tumor control. Dysfunction of T cells could also hamper T-cell responses which are required in immunotherapeutic strategies.

### 2.2. NK Cells

NK cells are important cellular mediators against virus-infected cells and tumor cells [36]. Less is known about NK cells in CLL compared to T cells; an overview of current knowledge can be found in Table 1.

High numbers of NK cells correlate with good prognosis in CLL, and NK cells were shown to be able to target CLL cells, highlighting their potential therapeutic activity [37,38]. However, CLL cells generally do not induce strong responses by autologous NK cells, indicating that CLL cells have mechanisms to evade NK cell recognition [39].

In contrast to T cells, NK cells do not recognize target cells via antigen-specific receptors, but instead rely on combined signaling via a variety of activating and inhibitory receptors to regulate their effector functions [40,41]. Despite their importance to initiate anti-tumor responses, reports on the expression levels of these receptors on NK cells in CLL, like NKp30, NKp46, DNAX accessory molecule-1 (DNAM-1), killer-cell immunoglobulin-like receptors (KIR), and CD16, are inconsistent [39,42,43,44,45]. CLL cells interfere with NK cell recognition mediated by these activating receptors; CLL cells induce downregulation of stimulating NK cell ligands on the cell surface, and produce soluble ligands to block receptor interaction, complicating recognition via natural cytotoxicity receptors [37,39,44,46,47]. Furthermore, proteins inhibiting NK cell responses, such as human leukocyte antigen(HLA)-E, HLA-G, and transforming growth factor beta (TGF-β) are upregulated by CLL cells [48,49,50,51].

It was long recognized that modulation of NK cell responses by CLL cells leads to reduced cytotoxic responses [39,42,43,44,45,52]. However, in contrast to the function of T cells, cytotoxicity of NK cells appears not intrinsically impaired in CLL. Treatment with anti-CD20 antibodies leads to targeting of CLL cells for antibody-dependent cellular cytotoxicity (ADCC) by NK cells [39,44,53]. Furthermore, taking NK cells out of the CLL environment restores their functionality [52]. This indicates that CLL cells disturb the balance between activating and inhibitory signaling, thereby hampering NK cell recognition, and escaping anti-tumor responses.

As NK cell function is intrinsically unaffected within the tumor micro-environment, and their recruitment via monoclonal antibodies readily induces therapeutic responses, NK cells are an attractive source of effector cells for immunotherapy in CLL.

## 3. Responses to Immunotherapy in CLL

### 3.1. Immune Checkpoint Blockade (ICB)

ICB revolutionized cancer therapy in recent years. ICB aims to boost anti-tumor responses by blocking inhibitory receptors (like PD-1 and CTLA-4) or their ligands (e.g., PD-L1) on immune cells or tumor cells. In hematologic malignancies, ICB showed clinical efficacy in multiple settings, most notably Hodgkin’s lymphoma, but also in follicular lymphoma and diffuse large B-cell lymphoma [8,9,54]. Since several reports describe the increased expression of PD-1 and CTLA-4 on T cells in CLL, and a PD-1/PD-L1 blockade induced therapeutic responses and recovery of T-cell function in a mouse model of CLL, blocking these receptors with monoclonal antibodies seemed a promising strategy [30,31,55,56]. However, early clinical trials showed disappointing results, with PD-1 blockade not leading to any objective responses, unless patients developed Richter’s transformation [9,57]. Combination therapy with BTK inhibitor ibrutinib and PD-1-blocking antibody nivolumab also did not show increased responses compared to ibrutinib monotherapy [58]. This indicates that the PD-1/PD-L1 axis is not the important mediator of immune dysfunction in CLL as was previously assumed, and blockade of PD-1/PD-L1 interaction as a single agent does not induce clinical responses. For an overview of ICB strategies in CLL, see Table 2. Other immune checkpoints are yet to be studied in the context of clinical trials in CLL.

Although most attention is focused on T cells to unleash anti-tumor responses with checkpoint inhibitors, NK cells also express similar inhibitory receptors. For example, blocking the inhibitory receptor NKG2A showed early clinical responses in head and neck cancer, inducing effector responses in both T and NK cells, and was tested in vitro for CLL [49,59,60]. Future studies aimed at increasing the activity of NK cells with immune checkpoint inhibitors would, therefore, be interesting.

Blocking a single inhibitory receptor on either T or NK cells is unlikely to substantially improve clinical responses. Combination therapy with agents that stimulate both T and NK cells is probably necessary to induce adequate immune responses and achieve significant clinical benefit.

### 3.2. Bi- and Tri-Specific Killer Engagers

In recent years, active recruitment of cytotoxic cells to tumor cells was achieved via the use of dual or triple targeting antibodies. These so-called bi- or tri-specific killer engagers (BiKE and TriKE) are constructs that consist of coupled antibody fragments that, on the one hand, recognize a tumor antigen, while, on the other hand, they bind to cytotoxic cells like T or NK cells, thereby inducing close contact between target and effector cells [61]. As these constructs can be adapted with relative ease, BiKE and TriKe constructs were manufactured in a multitude of ways, including with the use of different antibody fragments and linking formats [61]. BiKE constructs showed clinical activity in NHL like DLBCL and FL, in which 35–50% of patients showed response to treatment [11,12].

In CLL, the antibodies most studied are CD3xCD19 constructs, like blinatumomab (see Table 3) [62,63]. CD3xCD19 constructs are able to induce activation, differentiation, cytokine production, and expansion of T cells, and induce T-cell-mediated lysis of CLL cells in an autologous setting [62,63,64,65,66]. Interestingly, multiple reports describe strong synapse formation between autologous T cells and CLL target cells by CD3xCD19 constructs, indicating that targeting T cells with BiKEs overcomes the most significant functional defect in T cells of CLL patients [63,64]. Other molecular targets for CLL were also explored, like CD20 and receptor tyrosine kinase-like orphan receptor 1 (ROR1) [67,68,69]. Several trials using bi-specific antibodies to recruit T cells are currently ongoing in CLL.

NK cells were also targeted to tumor cells with similar bi-specific constructs. A tandem antibody targeting CD16xCD30 showed promising clinical efficacy in refractory Hodgkin’s lymphoma [72]. For CLL, a bi-specific construct was tested that targets CD19 while recruiting NK cells via ULBP2 (UL16 binding protein 2, the ligand of the cytotoxic NKG2D receptor) [70]. The same study showed activity of a tri-specific CD19xCD33xULBP2 construct, which targets tumor cells via two antigens while recruiting NK cells [70]. Interestingly, loss of one tumor antigen did not compromise the activity of the tri-specific antibody, showing that these dual-recognizing therapeutics can be used effectively to prevent tumor escape via antigen loss [70]. Autologous NK cell responses toward CLL were also induced by recruiting NK cells via the CD16 receptor using CD16xCD19 and CD16xCD19xCD22 constructs, which resulted in similar activation and induction of effector responses by NK cells [71]. In fact, responses induced by CD16xCD19xCD22 exceeded the response induced by rituximab, a monoclonal antibody to CD20 currently administered in first-line therapy for CLL [71]. This demonstrates that these constructs are more efficient in inducing NK cell effector responses via CD16 compared to monoclonal antibodies.

In addition, the CD16xCD19 construct was modified further to include the stimulatory IL-15 cytokine, which resulted in significantly higher levels of NK cell proliferation and killing [73]. These results highlight the vast amount of different strategies that can be exploited to improve these off-the-shelf products due to the relatively easy way in which different therapeutic molecules can be added or switched.

Although the use of BiKE and TriKE constructs is in an early phase of clinical testing for CLL, the in vitro results of these constructs demonstrate high potential for CLL therapy.

### 3.3. Chimeric Antigen Receptors

By far the best studied immunotherapeutic strategy for CLL in the last decade is the use of chimeric antigen receptor (CAR)-transduced T cells. Comprising an extracellular scFv fragment that recognizes a target antigen on tumor cells coupled to an intracellular tail containing T cell receptor (TCR) activation motifs and co-stimulatory domains, CAR constructs simultaneously redirect T cells toward tumor cells while inducing activating signaling and effector responses [74]. Since autologous T cells from the patient are used for treatment, CAR T-cell therapy does not lead to graft-versus-host responses. Furthermore, successful therapy leads to the establishment of memory T cells, which give prolonged protection from tumor relapse. Most trials studying CAR T-cell therapy in CLL used constructs recognizing CD19 on CLL tumor cells, while containing either a CD28 or 4-1BB co-stimulatory domain [75,76,77,78,79]. Despite a high response rate of similar constructs in ALL, the results of CAR T-cell therapy in CLL were relatively disappointing [14,80]. Although approximately 70% of CLL patients showed a response to therapy, the rate of complete remissions (CR) was much lower in CLL than in ALL (30% in CLL versus 70% in ALL) [78]. Since CLL patients that do achieve CR have an excellent prognosis and low rates of relapse, the current challenge for CAR T-cell therapy in CLL patients is to increase the rate of CRs [81]. For an overview of CAR constructs tested for CLL, see Table 4.

The immunomodulatory environment of CLL is assumed to have an important role in determining the response to CAR T-cell therapy. This is demonstrated by the fact that reducing the tumor load before transfusion of CAR T cells with lymphodepleting agents was shown to improve responses to CAR T-cell therapy [14,81]. Since CLL cells are able to modulate T-cell functionality, it was also recognized that intrinsic T-cell qualities may play a role. For example, the increased effector differentiation of T cells in CLL limits their proliferative capacity during the manufacturing process and after T-cell transfusion [86,87]. By retrospectively comparing T-cell infusion products from responders and non-responders, it was found that CAR T-cell products that were enriched for memory-cell populations with increased signal transducer and activator of transcription 3 (STAT3), cell cycle, and T-cell activation gene profiles, and with increased mitochondrial fitness yielded better responses to therapy [88,89]. In contrast, CAR T cells of non-responders were associated with increased effector differentiation and T-cell exhaustion profiles [88]. Furthermore, the strongest clinical correlates with CR are adequate CAR T-cell expansion and persistence after CAR T-cell infusion, two qualities of T cells that are mostly associated with memory populations [75,79,81]. The importance of memory cells in the transfusion product was demonstrated by a case report in which the integration of the CAR construct led to disruption of the *TET2* gene in transduced T cells (leading to a block in effector cell differentiation), which caused a clonal outgrowth of a highly effective CAR T-cell population within the patient, and ultimately led to the achievement of a delayed CR [90].

Similar to the BiKE and TriKE constructs, CARs can be modified relatively easily to increase functionality of the transduced T cells. For example, the CD28 and 4-1BB co-stimulatory domains of second-generation CARs lead to distinct differentiation states, with the CD28 domain inducing effector-memory T cells, while the 4-1BB domain leads to the formation of central memory cells, making the latter probably better suited for CLL therapy [91]. Third-generation CARs, which contain multiple co-stimulatory domains, are in development and being tested in the clinic [92]. Although most studies for CLL so far were performed with CARs targeting CD19, other constructs that target different antigens were described, including CD20, CD37, and malignancy-associated B-cell receptor chains [82,83,84,85]. Due to the frequent development of CD19-negative tumors after CAR T-cell therapy, targeting alternative antigens will be important to induce lasting anti-tumor responses. To reduce the odds of antigen escape, bi-specific CARs that target multiple antigens simultaneously were also developed, for example, toward CD19 and CD20 or CD19 and CD22, and are currently under clinical investigation [93]. The recent development of CAR constructs that divide T-cell activation and co-stimulatory domains across multiple antigen receptors with different specificity will enhance the possible strategies employed by bi-specific CAR constructs to effectively target CLL cells while reducing potential off-tumor side effects [94].

Despite the fact that NK cells retain cytotoxic function within the CLL micro-environment, the use of NK cells for CAR therapies is limited in CLL. This is probably related to several disadvantages NK cells have compared to T cells for CAR therapy. NK cell numbers are relatively low in peripheral blood, and NK cells are much more difficult to expand in vitro, making it hard to obtain enough autologous NK cells from each patient [95,96]. Furthermore, the almost complete lack of memory formation and the short lifespan of these innate effector cells hampers engraftment and longevity of any response, no matter how potent [95,96].

However, the use of NK cells does have several advantages over the use of T cells. Firstly, the risk of development of cytokine release syndrome (CRS), the most serious side effect of CAR T-cell therapy [97], is considerably lower when using NK cells due to the different cytokines they produce and their lower rate of expansion [95,96]. Secondly, potential side effects from CAR NK cell therapy would be relatively short-lived due to the reduced lifespan of the effector cells. Thirdly, CAR NK cells are not reliant only on the CAR construct to recognize tumor cells, but can also still be activated via other activating natural cytotoxicity receptors, and they remain effector mediators for ADCC and, therefore, show potential for combination therapy [40,41]. Finally, NK cells for CAR therapy could be used in an allogeneic setting, since transfer of allogeneic NK cells does not lead to graft-versus-host disease (GvHD) [98,99]. As multiple NK cell lines are available, like NK-92, NK cells have the potential to be an “off-the-shelf” therapeutic product, in contrast to autologous-based T-cell therapy.

CD19 CAR-transduced NK-92 cells showed cytotoxic efficacy toward multiple B-cell lines [100]. CAR NK and CAR NK-92 cells directed toward either CD19 and CD20 led to increased anti-tumor responses toward primary CLL cells, and the CD19-targeting CAR NK is currently under investigation in a clinical trial (NCT03056339) [101,102]. Allogeneic cord-blood NK cells were used as the source of effector cells in this trial, and, similarly to the TriKE construct mentioned earlier, CAR NK cells can be modified to express IL-15, which improves functionality [102]. CAR constructs with separated activation and co-stimulation domains spread over multiple specificities were transduced in NK-92 cells and showed tumor reactivity in vitro [103].

These data demonstrate the potential of NK cells in CAR therapy for CLL. Multiple clinical trials are currently ongoing with CAR NK cells for other B-cell malignancies, using similar CAR constructs that are under investigation for CAR T-cell therapy. Future studies should continue to include CLL patients to determine the effectivity of CAR NK cell therapy in this disease, especially if response rates to CAR T-cell therapy are difficult to improve.

### 3.4. Combination Strategies and the Role of Small-Molecule Inhibitors

In addition to their direct effects on CLL cells, several small-molecule inhibitors were reported to improve the function of non-malignant immune cells. Ibrutinib and venetoclax are two compounds associated with immune recovery in CLL patients [104,105]. Although there are less data on the effect of venetoclax, current results indicate that both ibrutinib and venetoclax treatment lead to a decrease in tumor-supportive T_fh_ and T_reg_ cells, while also reducing T-cell exhaustion [104,105]. Therefore, combination treatment of immunotherapy with small-molecule inhibitors may have beneficial effects, and multiple studies reported on these combinations.

Lenalidomide is an immunomodulatory drug that, despite inducing low levels of cell death in CLL cells, leads to therapeutic responses by restoring the function of the tumor micro-environment [106,107]. Lenalidomide was shown to restore the defect in synapse formation of T cells, and enhance the cytotoxic function of NK cells [31,108]. Treatment with lenalidomide is associated with significant toxicity; however, due to the high response rate (ORR of 64% in relapsed patients in combination with rituximab [109,110]), it was included in several clinical trials for CLL. However, lenalidomide is not yet approved for CLL and is not used in routine clinical practice.

Phosphoinositide 3-kinase delta (PI3Kδ) inhibition by idelalisib does induce direct cell death in CLL cells, but inhibition of PI3Kδ also affects T cells. T_reg_ cells in particular were found to be sensitive to idelalisib treatment, but effector T-cell differentiation and functionality were also affected [111,112]. The reduction of T_reg_ numbers and function is considered to be responsible for the long-term auto-immune mediated toxicity of idelalisib treatment in CLL patients, especially colitis, pneumonitis, and hepatitis [113]. A recent study used idelalisib treatment during the production of CAR T cells to block effector T-cell differentiation, which resulted in an increase in memory cells and was associated with better CAR T-cell responses in mouse models [114]. This shows the potential benefit of using small-molecule inhibitors during the preparation of immunotherapeutic effectors in the laboratory phase, without the potential toxicity of administering these drugs in vivo.

Another drug with well-documented effects on non-malignant immune cells is ibrutinib [115]. Ibrutinib affects T cells due to the off-target inhibition of IL2-inducible T cell kinase (ITK). After ibrutinib treatment, T cells show decreased expression of the inhibitory receptors PD-1 and CTLA-4, T_reg_ numbers are decreased, and anti-tumor T_H_1 responses are increased [104,116]. Although reports on overall T-cell numbers are inconsistent, TCR diversity increased after ibrutinib treatment, indicating restoration of T-cell immunity [117]. T cells from ibrutinib-treated patients showed improved responses to both CD3xCD19 and ROR1xCD3 bi-specific antibodies ex vivo. Ibrutinib pre-treatment increased synapse formation and cytotoxicity toward CLL cells in vitro, demonstrating a potential benefit of bi-specific antibodies and ibrutinib in combinatory strategies [64,68]. Since these effects were also seen toward ibrutinib-resistant CLL cells, the beneficial effects of ibrutinib seem to be directly on T cells themselves [64]. Ibrutinib showed similar beneficial effects in CAR T-cell therapy. Ibrutinib-treated T cells showed increased proliferation and expansion in vivo, reduced expression of inhibitory molecules, enhanced engraftment, and increased anti-tumor activity [77,78]. In a phase 1/2 trial, pre-treatment with ibrutinib for several weeks increased the response rates to CAR T-cell therapy in CLL patients to 88% (compared to 56% in the ibrutinib-naïve cohort), demonstrating the clinical value of combination treatment [118,119]. At this stage, it is still unclear whether all beneficial effects of ibrutinib are caused directly via T-cell modulation, or whether additional indirect effects via CLL tumor cells or the tumor micro-environment are responsible for the increased response to therapy [81].

One aspect that may currently not receive sufficient attention is the potential of immunotherapy as consolidation therapy in CLL. As disappearance of the tumor clone ameliorates immunosuppression, effective treatment with small-molecule inhibitors like ibrutinib and venetoclax could open the door for improved responses to immunotherapy in tumor remission. This strategy could, on the one hand, lead to long-lasting remission and protection from relapse by immunotherapeutics, while, on the other hand, it could give the opportunity to stop treatment with small-molecule inhibitors, leading to decreased development of tumor escape mechanisms and possibly a reduction in treatment costs. An important issue in the development of such combined strategies is the timing of administering immunotherapy. Finding the right balance between inducing sufficient remission to restore anti-tumor responses while still having enough antigen present to induce effective immune responses should be addressed in clinical studies.

Taken together, small-molecule inhibitors may play an important role in future immunotherapy in CLL by modulating tumor cells, the tumor micro-environment, and/or the cytotoxic effector cells. Future studies should further investigate the influence of small-molecule inhibitors on the non-malignant immune system, and evaluate the potential of different drug combinations for successful CLL therapy.

## 4. Concluding Remarks

As the requirements for optimal activity of immunotherapy become clear, response rates of CLL patients will continue to improve. Although T cells are currently on the forefront and were shown to be able to induce complete responses in some patients, CLL patients may benefit from the recruitment of NK cells into immunotherapeutic regimens, as these cells were shown to be less functionally impaired compared to autologous T cells. Furthermore, long-lasting immune protection by immunotherapy may also be induced by strategies combining treatment with small-molecule inhibitors, followed by immunotherapy as a consolidation treatment.

The plethora of different strategies available fuels the belief that both T and NK cells can be used in an effective way to overcome the immunosuppressive tumor micro-environment and induce lasting therapeutic responses in, and possibly even cure CLL.

## Figures and Tables

**Table 1:** Overview of permutations of T and natural killer (NK) cells in previously untreated chronic lymphocytic leukemia (CLL) patients.

	CD4 T Cells	CD8 T Cells	NK Cells
Absolute numbers	Increased	Increased	Increased
Differentiation	Naïve↓ Effector ↑ T_H_1 ↑ T_H_2 T_fh_ ↑ T_reg_ ↑	Naïve↓ Effector ↑	Increased maturation
Cytokine production	High	High	Low
Proliferation	Low	Low	Low
Cytotoxicity	/	Low	Natural cytotoxicity: low ADCC: normal
Exhaustion markers	High	High	Inconsistent

CD—cluster of differentiation; T_H_—T helper cell; T_fh_—T follicular helper cells; T_reg_—T regulatory cell; ADCC—antibody-dependent cellular cytotoxicity. ↑—increased in CLL; ↓—decreased in CLL.

**Table 2:** Overview of immune checkpoint inhibitors tested in CLL.

Treatment	Target	Phase	# patients	ORR	Reference
Nivolumab	PD-1	2	3	0	[9]
Pembrolizumab	PD-1	2	16	0	[57]
Nivolumab + ibrutinib	PD-1	2	36	61 *	[58]
**In vitro studies**					
Anti-TIGIT-Fc	TIGIT	-	-	-	[23]
Monalizumab	CD94/NKG2A	-	-	-	[49]

#—number of patients; ORR–objective response rate. * ORR is similar to ibrutinib monotherapy.

**Table 3:** Overview of bi- and tri-specific killer engagers tested in vitro in CLL.

Construct Type	Target	Effector	Reference
Bi-specific single-chain antibody	CD19xCD3	T cells	[62,63]
Bi-specific single-chain Fc-Fv	CD19xCD3	T cells	[64]
DART	CD19xCD3	T cells	[65,66]
DART	CD20xCD3	T cells	[67]
Bi-specific single-chain antibody	ROR1xCD3	T cells	[68]
Bi-specific single-chain Fc-Fv	ROR1xCD3	T cells	[69]
Bi-specific single-chain antibody	CD19xCD19xNKG2D	NK cells	[70]
Tri-specific single-chain antibody	CD19xCD33xNKG2D	NK cells	[70]
Bi-specific single-chain antibody	CD19xCD16	NK cells	[71]
Tri-specific single-chain antibody	CD19xCD22xCD16	NK cells	[71]

DART = dual—affinity re—targeting antibody.

**Table 4:** Overview of chimeric antigen receptor (CAR) constructs tested in CLL and discussed in this review. CR—complete remission.

Target	Co-stimulation Domain	Phase	No. of patients	% ORR/CR	Reference
CD19	4-1BB	1/2	24	71/21	[75]
CD19	4-1BB	1/2	14	57/29	[76]
CD19	CD28z	1	16	38/12	[78]
BCR κ chains	CD28z	1	2	0/0	[82]
**In vitro studies**					
CD20	CD28z	/	/	/	[83]
CD37	4-1BB	/	/	/	[84]
BCR Fc µ chains	CD28z	/	/	/	[85]

For a comprehensive review on clinical response rates to these CAR constructs in CLL, see References [14,80]. BCR—B cell receptor.
